# *cis*-Dichloridobis[2-(hydroxy­meth­yl)tetra­hydro­furan-κ^2^ *O*,*O*′]manganese(II)

**DOI:** 10.1107/S1600536808009549

**Published:** 2008-04-10

**Authors:** Lucjan B. Jerzykiewcz, Józef Utko, Piotr Sobota

**Affiliations:** a Faculty of Chemistry, University of Wrocław, 14. F. Joliot Curie, 50-383 Wrocław, Poland

## Abstract

The structure of the title compound, [MnCl_2_(C_5_H_10_O_2_)_2_], was solved from low-temperature data collected at 100 (2) K. The asymmetric unit contains one half-mol­ecule with the Mn^II^ ion located on a twofold axis. A distorted octa­hedral environment around the Mn atom is formed by two ether and two hydroxyl O atoms of two 2-(hydroxy­methyl)tetra­hydro­furan ligands, and by two chloride ions. The chelating tetra­hydro­furan ligands, which form five-membered rings, are *cis* oriented. The crystal structure is stabilized by hydrogen bonding between the coordinated OH groups and the chloride ions.

## Related literature

For general background, see: Bradley (1989 ▶); Hubert-Pfalzgraf (1998 ▶); Jerzykiewicz *et al.* (1997 ▶, 2007*a* ▶,*b* ▶); Janas *et al.* (1997 ▶, 1999 ▶); Sobota *et al.* (1998*a* ▶,*b* ▶, 2000*a* ▶,*b* ▶); Utko *et al.* (2003 ▶)·For related compounds, see: Wu *et al.* (2004 ▶); Lumme & Lindell (1988 ▶); Choudhury *et al.* (2006 ▶); Yang *et al.* (2003 ▶).
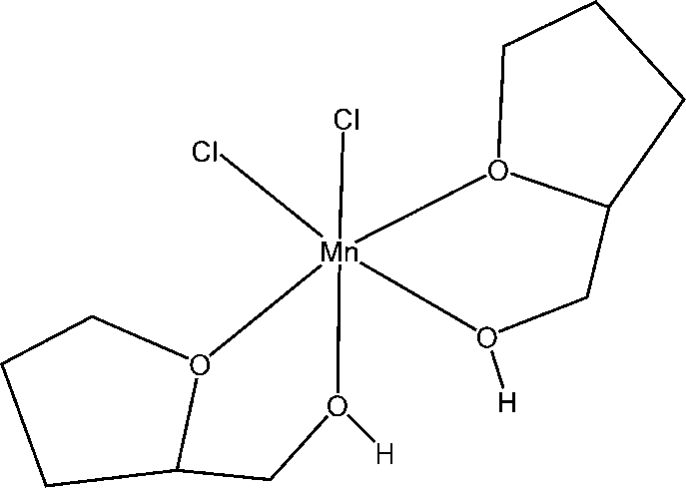


## Experimental

### 

#### Crystal data

[MnCl_2_(C_5_H_10_O_2_)_2_]
*M* *_r_* = 330.10
Monoclinic, 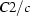

*a* = 17.463 (3) Å
*b* = 6.171 (2) Å
*c* = 13.159 (3) Å
β = 100.24 (2)°
*V* = 1395.5 (6) Å^3^
*Z* = 4
Mo *K*α radiation
μ = 1.33 mm^−1^
*T* = 100 (2) K
0.33 × 0.21 × 0.18 mm

#### Data collection

Kuma KM-4 CCD κ-axis diffractometer
Absorption correction: analytical (*CrysAlis CCD*; Oxford Diffraction, 2006 ▶) *T*_min_ = 0.721, *T*_max_ = 0.818
7753 measured reflections
1757 independent reflections
1667 reflections with *I* > 2σ(*I*)
*R*_int_ = 0.034

#### Refinement

*R*[*F* ^2^ > 2σ(*F* ^2^)] = 0.025
*wR*(*F* ^2^) = 0.057
*S* = 1.13
1757 reflections
91 parameters
H atoms treated by a mixture of independent and constrained refinement
Δρ_max_ = 0.36 e Å^−3^
Δρ_min_ = −0.32 e Å^−3^

### 

Data collection: *CrysAlis CCD* (Oxford Diffraction, 2006 ▶); cell refinement: *CrysAlis RED* (Oxford Diffraction, 2006 ▶); data reduction: *CrysAlis RED*; program(s) used to solve structure: *SHELXTL* (Sheldrick, 2008 ▶); program(s) used to refine structure: *SHELXTL*; molecular graphics: *DIAMOND* (Brandenburg, 2007 ▶); software used to prepare material for publication: *SHELXTL*, *PLATON* (Spek, 2003 ▶), *enCIFer* (Allen *et al.*, 2004 ▶) and *publCIF* (Westrip, 2008 ▶).

## Supplementary Material

Crystal structure: contains datablocks I, global. DOI: 10.1107/S1600536808009549/kp2165sup1.cif

Structure factors: contains datablocks I. DOI: 10.1107/S1600536808009549/kp2165Isup2.hkl

Additional supplementary materials:  crystallographic information; 3D view; checkCIF report

## Figures and Tables

**Table:** 

Mn—Cl1	2.459 (1)
Mn—O10	2.222 (2)
Mn—O11	2.222 (2)

**Table:** 

Cl1—Mn—O10	94.26 (3)
Cl1—Mn—O11	89.57 (3)
Cl1—Mn—Cl1^i^	99.60 (2)
Cl1—Mn—O10^i^	93.03 (3)
Cl1—Mn—O11^i^	164.10 (3)
O10—Mn—O11	73.22 (4)
O10—Mn—O10^i^	168.70 (4)
O10—Mn—O11^i^	98.24 (4)
O11—Mn—O11^i^	84.69 (4)

Symmetry code: (i) 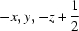.

**Table 2:** Hydrogen-bond geometry (Å, °)

*D*—H⋯*A*	*D*—H	H⋯*A*	*D*⋯*A*	*D*—H⋯*A*
O11—H11⋯Cl1^ii^	0.79 (2)	2.26 (2)	3.021 (2)	164 (2)

Symmetry code: (ii) 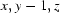.

**Fig. 1.**
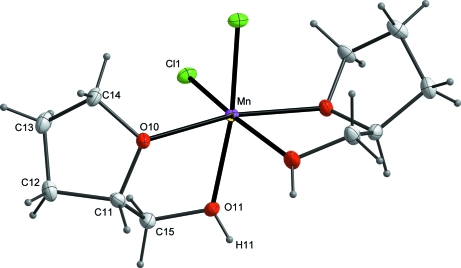
A view of the title compound with the atom numbering scheme. Displacement ellipsoids for non-H atoms are drawn at the 50% probability level. Mn(II) is located at the two-fold axes.

**Fig. 2.**
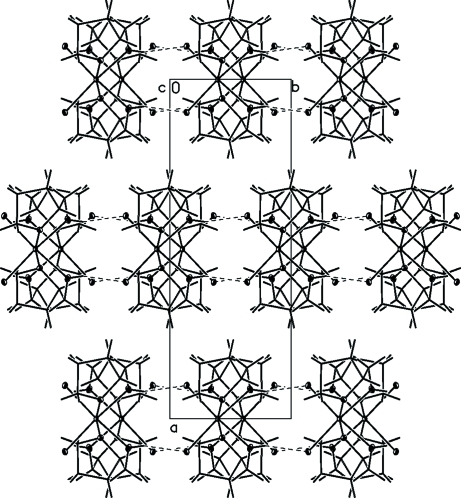
The packing of the title compound, viewed down the c axis, showing one layer of molecules connected by O—H···Cl hydrogen bonds (dashed lines).

## supplementary crystallographic information

### Comment

The investigation presented in this work is a part of our research project concerning complexes with *O*,*O'*–bifunctional ligands (Jerzykiewicz *et al.*, 1997; Janas *et al.*, 1997; Sobota *et al.*, 1998*a*; Sobota *et al.*, 1998*b*; Janas *et al.*, 1999; Sobota *et al.*, 2000*a*; Sobota *et al.*, 2000*b*; Utko *et al.*, 2003, Jerzykiewicz *et al.*, 2007*a*; Jerzykiewicz *et al.* 2007*b*). The use of chelating alkoxides can provide new compounds which are potential candidates for both sol-gel and metal-organic chemical vapour (MOCV) conversion of the precursor into the ceramic materials (Hubert-Pfalzgraf, 1998; Bradley, 1989). In this paper we describe the structure of a monomeric manganese(II) alkoxide complex: Mn(thffoH)_2_Cl_2_ (thffoH – tetrahydrofurfuryl alcohol) (Fig. 1). The Mn^II^ atom located at  the special position (0, y, 1/4) on the two-fold axes displays a slightly distorted octahedral geometry (Table 1). The thffoH molecules bond to Mn atom as bidentate ligands through O11 hydroxyl group and  O10 of ether group close two five-membered rings. The hydroxyl groups are *cis* arranged, whereas ether oxygen atoms of chelating ligands are situated *trans*. The coordination sphere of metal ion is completed by the Cl^-^ ions, which are in *cis*-positions. In contrast to other structures with *O,O'*–functional ligand MnBr_2_(MeOH)(Hmepap) (where mepma = *N*–(2–methoxyethyl)–*N*–(pyridin–2–ylmethyl)amine) (Wu *et al.*, 2004) and Mn_4_Cl_4_(OCH_2_CH_2_OCH_3_)_4_(EtOH)_4_ (Jerzykiewicz *et al.*, 2007*a*) the lengths of Mn–O(ether) and Mn–*O*(hydroxyl) bonds do not differ significantly. The Mn–Cl bond length of 2.459 (1) Å is similar to corresponding bonds distances in other monomeric octahedral manganese (II) compounds with *cis*–Cl atoms Mn(2,2'-bpy)_2_(C1)_2_ (where 2,2'-bpy = 2,2'-bipyridine) (Lumme & Lindell, 1988), Mn(2,2'-bpy)_2_(C1)_2_^.^SC(NH_2_)_2_ (Choudhury *et al.*, 2006), MnCl_2_(HL)_2_ (where HL = *N*–(3–chlorophenyl)pyridine–2–carboxamide) (Yang *et al.*, 2003). The tetrahydrofouran ring adopts an envelope conformation. The whole structure is held together by intermolecular hydrogen bonds of O–H···Cl type (Table 2, Fig. 2).

### Experimental

The air- and moisture-sensitive title compound was prepared under dried N_2_. A mixture of 1.26 g (10 mmol) MnC1_2_ and 1.93 cm^3^ (20 mmol) tetrahydrofurfuryl alcohol (thffoH, Aldrich) in 15 mL of absolute ethanol was refluxed for 50 min, and the resulting precipitate was filtered, washed with ethanol, dried and recrystallized from ethanol.

### Refinement

Carbon bonded hydrogen atoms were included in calculated positions and refined in the riding mode using *SHELXTL* default parameters. The remaining H atoms were located in a difference map and refined freely.

### Crystal data

**Table:**

[MnCl_2_(C_5_H_10_O_2_)_2_]	*F*_000_ = 684
*M**_r_* = 330.10	*D*_x_ = 1.571 Mg m^−^^3^
Monoclinic, *C*2/*c*	Mo *K*α radiation λ = 0.71073 Å
Hall symbol: -C 2yc	Cell parameters from 4812 reflections
*a* = 17.463 (3) Å	θ = 3–29º
*b* = 6.171 (2) Å	µ = 1.33 mm^−^^1^
*c* = 13.159 (3) Å	*T* = 100 (2) K
β = 100.24 (2)º	Block, colorless
*V* = 1395.5 (6) Å^3^	0.33 × 0.21 × 0.18 mm
*Z* = 4	

### Data collection

**Table:**

Kuma KM-4 CCD κ-axis diffractometer	1757 independent reflections
Radiation source: fine-focus sealed tube	1667 reflections with *I* > 2σ(*I*)
Monochromator: graphite	*R*_int_ = 0.034
*T* = 100(2) K	θ_max_ = 28.5º
ω scans	θ_min_ = 3.6º
Absorption correction: analytical(CrysAlis CCD; Oxford Diffraction, 2006)	*h* = −23→23
*T*_min_ = 0.721, *T*_max_ = 0.818	*k* = −8→8
7753 measured reflections	*l* = −11→17

### Refinement

**Table:**

Refinement on *F*^2^	Secondary atom site location: difference Fourier map
Least-squares matrix: full	Hydrogen site location: inferred from neighbouring sites
*R*[*F*^2^ > 2σ(*F*^2^)] = 0.025	H atoms treated by a mixture of independent and constrained refinement
*wR*(*F*^2^) = 0.057	*w* = 1/[σ^2^(*F*_o_^2^) + (0.0255*P*)^2^ + 0.9537*P*] where *P* = (*F*_o_^2^ + 2*F*_c_^2^)/3
*S* = 1.13	(Δ/σ)_max_ < 0.001
1757 reflections	Δρ_max_ = 0.36 e Å^−^^3^
91 parameters	Δρ_min_ = −0.32 e Å^−^^3^
Primary atom site location: structure-invariant direct methods	Extinction correction: none

### Special details

**Table:**

Geometry. All e.s.d.'s (except the e.s.d. in the dihedral angle between two l.s. planes) are estimated using the full covariance matrix. The cell e.s.d.'s are taken into account individually in the estimation of e.s.d.'s in distances, angles and torsion angles; correlations between e.s.d.'s in cell parameters are only used when they are defined by crystal symmetry. An approximate (isotropic) treatment of cell e.s.d.'s is used for estimating e.s.d.'s involving l.s. planes.
Refinement. Refinement of *F*^2^ against ALL reflections. The weighted *R*-factor *wR* and goodness of fit *S* are based on *F*^2^, conventional *R*-factors *R* are based on *F*, with *F* set to zero for negative *F*^2^. The threshold expression of *F*^2^ > σ(*F*^2^) is used only for calculating *R*-factors(gt) *etc*. and is not relevant to the choice of reflections for refinement. *R*-factors based on *F*^2^ are statistically about twice as large as those based on *F*, and *R*- factors based on ALL data will be even larger.

### Fractional atomic coordinates and isotropic or equivalent isotropic displacement parameters (Å^2^)

**Table:**

	*x*	*y*	*z*	*U*_iso_*/*U*_eq_	
Mn	0.0000	0.60449 (4)	0.2500	0.01153 (8)	
Cl1	0.097635 (19)	0.86166 (5)	0.33711 (2)	0.01677 (9)	
O10	0.05616 (5)	0.56904 (15)	0.11203 (7)	0.01400 (19)	
O11	0.08645 (6)	0.33832 (17)	0.28441 (8)	0.0203 (2)	
H11	0.0799 (13)	0.215 (4)	0.2945 (16)	0.035 (6)*	
C11	0.11816 (8)	0.4088 (2)	0.11569 (11)	0.0158 (3)	
H11A	0.0972	0.2725	0.0801	0.017 (4)*	
C12	0.17872 (8)	0.5110 (2)	0.05927 (11)	0.0192 (3)	
H12A	0.2323	0.4768	0.0946	0.021 (4)*	
H12B	0.1719	0.4602	−0.0132	0.028 (5)*	
C13	0.16192 (8)	0.7537 (2)	0.06439 (12)	0.0203 (3)	
H13A	0.1872	0.8166	0.1311	0.029 (5)*	
H13B	0.1794	0.8332	0.0073	0.033 (5)*	
C14	0.07401 (8)	0.7568 (2)	0.05337 (11)	0.0171 (3)	
H14A	0.0562	0.8921	0.0822	0.028 (5)*	
H14B	0.0490	0.7444	−0.0200	0.024 (5)*	
C15	0.14969 (8)	0.3643 (2)	0.22866 (11)	0.0183 (3)	
H15A	0.1817	0.2310	0.2352	0.024 (4)*	
H15B	0.1832	0.4862	0.2584	0.017 (4)*	

### Atomic displacement parameters (Å^2^)

**Table:**

	*U*^11^	*U*^22^	*U*^33^	*U*^12^	*U*^13^	*U*^23^
Mn	0.01366 (14)	0.00883 (13)	0.01296 (14)	0.000	0.00471 (10)	0.000
Cl1	0.01853 (17)	0.01165 (15)	0.01927 (17)	−0.00251 (11)	0.00099 (12)	−0.00065 (11)
O10	0.0142 (4)	0.0143 (4)	0.0147 (4)	0.0012 (3)	0.0060 (4)	0.0023 (3)
O11	0.0236 (5)	0.0134 (5)	0.0276 (6)	0.0050 (4)	0.0145 (4)	0.0082 (4)
C11	0.0138 (6)	0.0133 (6)	0.0211 (7)	0.0007 (5)	0.0055 (5)	−0.0028 (5)
C12	0.0154 (6)	0.0251 (7)	0.0187 (7)	0.0004 (5)	0.0073 (5)	0.0007 (5)
C13	0.0153 (6)	0.0231 (7)	0.0224 (7)	−0.0042 (5)	0.0027 (5)	0.0073 (5)
C14	0.0166 (6)	0.0183 (6)	0.0167 (6)	−0.0014 (5)	0.0041 (5)	0.0070 (5)
C15	0.0161 (6)	0.0178 (6)	0.0226 (7)	0.0042 (5)	0.0076 (5)	0.0056 (5)

### Geometric parameters (Å, °)

**Table:**

Mn—Cl1	2.459 (1)	C13—C14	1.516 (2)
Mn—O10	2.222 (2)	O11—H11	0.78 (2)
Mn—O11	2.222 (2)	C11—H11A	1.00
Mn—Cl1^i^	2.459 (1)	C12—H12A	0.99
Mn—O10^i^	2.222 (2)	C12—H12B	0.99
Mn—O11^i^	2.222 (2)	C13—H13A	0.99
O10—C11	1.461 (2)	C13—H13B	0.99
O10—C14	1.456 (2)	C14—H14A	0.99
O11—C15	1.439 (2)	C14—H14B	0.99
C11—C12	1.532 (2)	C15—H15A	0.99
C11—C15	1.516 (2)	C15—H15B	0.99
C12—C13	1.530 (2)		
			
Cl1—Mn—O10	94.26 (3)	O10—C14—C13	104.34 (10)
Cl1—Mn—O11	89.57 (3)	O11—C15—C11	110.01 (11)
Cl1—Mn—Cl1^i^	99.60 (2)	O10—C11—H11A	110
Cl1—Mn—O10^i^	93.03 (3)	C12—C11—H11A	110
Cl1—Mn—O11^i^	164.10 (3)	C15—C11—H11A	110
O10—Mn—O11	73.22 (4)	C11—C12—H12A	111
Cl1^i^—Mn—O10	93.03 (3)	C11—C12—H12B	111
O10—Mn—O10^i^	168.70 (4)	C13—C12—H12A	111
O10—Mn—O11^i^	98.24 (4)	C13—C12—H12B	111
Cl1^i^—Mn—O11	164.10 (3)	H12A—C12—H12B	109
O10^i^—Mn—O11	98.24 (4)	C12—C13—H13A	111
O11—Mn—O11^i^	84.69 (4)	C12—C13—H13B	111
Cl1^i^—Mn—O10^i^	94.26 (3)	C14—C13—H13A	111
Cl1^i^—Mn—O11^i^	89.57 (3)	C14—C13—H13B	111
O10^i^—Mn—O11^i^	73.22 (4)	H13A—C13—H13B	109
Mn—O10—C11	118.15 (8)	O10—C14—H14A	111
Mn—O10—C14	121.45 (8)	O10—C14—H14B	111
C11—O10—C14	109.20 (10)	C13—C14—H14A	111
Mn—O11—C15	111.64 (8)	C13—C14—H14B	111
Mn—O11—H11	129.6 (17)	H14A—C14—H14B	109
C15—O11—H11	110.0 (17)	O11—C15—H15A	110
C12—C11—C15	112.84 (12)	O11—C15—H15B	110
O10—C11—C12	106.07 (10)	C11—C15—H15A	110
O10—C11—C15	107.03 (11)	C11—C15—H15B	110
C11—C12—C13	103.14 (11)	H15A—C15—H15B	108
C12—C13—C14	101.94 (11)		
			
Cl1—Mn—O10—C11	85.96 (8)	Mn—O10—C11—C15	−21.30 (12)
Cl1—Mn—O10—C14	−53.90 (9)	C14—O10—C11—C12	2.38 (14)
O11—Mn—O10—C11	−2.30 (8)	C14—O10—C11—C15	123.09 (11)
O11—Mn—O10—C14	−142.16 (10)	Mn—O10—C14—C13	117.39 (10)
Cl1^i^—Mn—O10—C11	−174.18 (8)	C11—O10—C14—C13	−25.61 (13)
Cl1^i^—Mn—O10—C14	45.96 (9)	Mn—O11—C15—C11	−48.82 (11)
O11^i^—Mn—O10—C11	−84.18 (9)	O10—C11—C12—C13	21.45 (14)
O11^i^—Mn—O10—C14	135.96 (9)	C15—C11—C12—C13	−95.43 (13)
Cl1—Mn—O11—C15	−67.02 (8)	O10—C11—C15—O11	44.63 (13)
O10—Mn—O11—C15	27.56 (8)	C12—C11—C15—O11	160.94 (10)
O10^i^—Mn—O11—C15	−160.02 (8)	C11—C12—C13—C14	−36.08 (14)
O11^i^—Mn—O11—C15	127.83 (9)	C12—C13—C14—O10	38.10 (13)
Mn—O10—C11—C12	−142.01 (9)		

Symmetry codes: (i) −*x*, *y*, −*z*+1/2.

### Hydrogen-bond geometry (Å, °)

**Table:**

*D*—H···*A*	*D*—H	H···*A*	*D*···*A*	*D*—H···*A*
O11—H11···Cl1^ii^	0.79 (2)	2.26 (2)	3.021 (2)	164 (2)

Symmetry codes: (ii) *x*, *y*−1, *z*.
